# Trends in Positioning for Robotic Prostatectomy: Results From a Survey of the Endourological Society

**DOI:** 10.7759/cureus.12628

**Published:** 2021-01-11

**Authors:** George Wayne, Jeff Wei, Elias Atri, Vivian Wong, Maurilio Garcia-Gil, Jorge Pereira, Alan M Nieder, Akshay Bhandari

**Affiliations:** 1 Urology, Mount Sinai Medical Center, Miami Beach, USA; 2 Urology, Florida International University Herbert Wertheim College of Medicine, Miami, USA

**Keywords:** robotics, prostate cancer, laparoscopy, lithotomy

## Abstract

Purpose: most robot-assisted laparoscopic prostatectomies (RALP) are performed with the patient in lithotomy, carrying risks of positioning-related complications. Newer robot models have allowed for supine positioning, potentially avoiding these pitfalls. We gauged the current sentiment on patient positioning among surgeons who perform robot-assisted surgery.

Methods: we surveyed members of the Endourological Society regarding their practice settings and their opinions on positioning for robot-assisted laparoscopic prostatectomy. Summary statistics were reviewed and data were analyzed using chi-square tests and t-tests.

Results: our survey had 92 eligible respondents. The majority were fellowship-trained, with 51% trained in robotics and 57% practicing in the U.S. with a mean of 13 years of practice. Most were working in an academic setting (69%) and performing at least 25 robotic prostatectomies yearly. 28 respondents used the Intuitive Surgical Inc. da Vinci® Xi™ exclusively (30%), and nearly two-thirds used it sometimes. Although 54% of respondents considered using supine positioning, less than half of these surgeons used it regularly, while 75% overall preferred lithotomy. A majority attributed this choice to surgical team familiarity with lithotomy positioning. Surgeons in the U.S. and those using the da Vinci® Xi™ were more likely to consider supine positioning.

Conclusions: lithotomy position is the standard for RALP procedures; nonetheless, it poses significant risks that might be avoided with supine positioning. Our survey suggests that, although supine positioning has been considered, it has not gained momentum in practice. Addressing factors of inertia in training practices and one’s surgical team might allow for novel and safer approaches.

## Introduction

Robot-assisted laparoscopic prostatectomy (RALP) has seen widespread and rapid adoption in the last decade with RALP becoming the most common approach to radical prostatectomy. The rates of radical prostatectomies increased by 60% between the years 2005 and 2008 [1]. Recent data indicates that 67% to 85% of radical prostatectomies (RPs) were done robotically [1]. RPs gained popularity quickly after their initial introduction in 2000 [1]. Despite higher costs, RALP continues to expand in adoption, possibly due to perceived lower morbidity [2]. At this time, the most common approach remains transperitoneal with the patient in the lithotomy position [3].

Lithotomy was initially used for robot access when RALP was first adopted, without significant evolution since [4]. Lithotomy is associated with a significant risk of neuropathy, venous thrombosis, compartment syndrome, and possible anesthesia complications, especially in longer cases [5]. Choice of surgical positioning has a significant effect on the patient - both physiologically and economically. The cardiovascular and respiratory effects of lithotomy and steep Trendelenberg positioning are directly related to positional and gravitational stress [6]. The raised orientation of the diaphragm results in a significant decrease in pulmonary compliance which can cause edema of the upper airway and brain. In addition, normal compensatory methods such as hyperventilation are more stressful to the body due to the gravitational effect of the Trendelenberg position.

The etiology of nerve injuries is associated with stretch and compressive forces of lithotomy, even when using devices intended to prevent patient movement during surgery. Warner et al. retrospectively reviewed 198,461 patients who underwent surgical procedures performed in the lithotomy position in a period of 34 years at the Mayo Clinic [5]. Persistent neuropathies were seen in 55 cases (rate of 1 per 3,608). Risk factors included operative time over four hours, body mass index of 20 or less, and smoking within 30 days before the procedure. The authors note the reduction of time in lithotomy position may be particularly worthwhile for patients with these risk factors [5]. In a follow-up study, the authors found that two hours of lithotomy positioning significantly raised the risk of lower extremity neuropathy [7]. Other rare complications such as femoral neuropathy and compartment syndrome have also been described [8, 9]. Anema et al. evaluated 185 patients who underwent urethral reconstruction in the lithotomy position. Of these patients, 18 (10%) incurred position-related complications, including compartment syndrome, rhabdomyolysis, and neurapraxia. Longer procedures had higher complication rates. When penile skin flap procedures for flap harvesting were begun in the supine position before repositioning into lithotomy, the risk of complications was virtually eliminated [10]. Although lacking some external validity, such data from other urologic procedures ought to be considered when planning robotic prostatectomy.

Although these complications are rare, they may carry significantly increased costs and longer hospital stays. Wen et al. in 2014 looked at patients undergoing RALP and found that positioning complications increased inpatient costs by 400% [11]. They noted the main driver of increased costs related to robotic prostatectomy was not the procedure itself but rather positioning complications and other comorbidities [11]. Such events increased the odds of having a longer hospital stay by three times [11]. Combined with the tendency for underreporting complications, this emphasizes the impact of any position-related complications on the economic burden. Further efforts to minimize a small but significant number of complications have the potential to significantly impact total costs and overall length of stay.

A simple avenue to avoid complications associated with the physiologic stress of steep lithotomy would be side docking in the supine position, which has been described extensively in the literature. Even with the older da Vinci® Si™ system, several studies indicate the feasibility of supine positioning using this technique [12,13]. Side docking, as shown in previous urologic and gynecologic studies, potentially improves access to the perineum and decreases arm collision, improving assistant access while decreasing set up time [14,15,16,17]. Neurological complications of the lower limbs may be prevented by the use of side-docking techniques [18]. In a randomized control trial with a cohort of 120 patients undergoing radical prostatectomy, none of those who underwent side docking had lower limb neurologic defects while 10% (six of 60) of patients in traditional docking with steep Trendelenburg did [18]. With side docking, there are no significant changes necessary for port placement or overall surgical technique [18]. In the current era of the Xi robot, which features an extendable boom arm as well as longer instrument working arms, side-docking should allow for an easier transition to supine positioning [18]. The aim of this survey was to gauge the current sentiment with respect to patient positioning among active robotic surgeons and identify any targets for improvement.

## Materials and methods

We surveyed members of the Endourological Society regarding their practice settings as well as their opinions of positioning for robot-assisted laparoscopic prostatectomy. The survey was sent to the listserv of the Journal of Endourology via email in September 2018 which included approximately 2500 addresses. Responses were collected via SurveyMonkey® survey hosting service using a combination of free-response and multiple-choice questions (please see the Appendices section). The survey was anonymous. Demographic data on years in practice, fellowship training, and practice setting were queried. Specific questions relating to robotic prostatectomies included: the model of the robot, positioning for RALPs, level of exposure, and reasoning for lithotomy. Two e-mail reminders were sent out for survey completion. A copy of the survey is included in the appendices. Summary statistics were reviewed and data were analyzed using chi-squared test and t-test.

## Results

**Figure 1:**
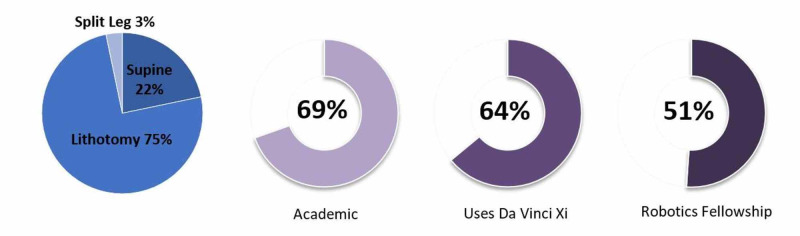
Respondent demographics and survey results

One hundred surgeons elected to participate in our survey. Eight respondents provided incomplete information and were excluded from the analysis. The remainder represented 52 responses from the U.S., 14 from various parts of Europe, 11 from Africa and the Middle East, nine from South America, and six from countries in Asia. The overall response rate was approximately 4%. Of these, 51% completed a fellowship in Robotic Surgery, and 69% practiced in an academic setting (Figure 1). They were on average 13.3 years into independent practice (median 11.5, range 1-38). Most were performing at least 25 robotic prostatectomies yearly (70%). Only 30% used the Intuitive Surgical Inc. da Vinci® Xi™ exclusively, though 64% were using it at least some of the time.

With respect to positioning preferences, 75% of the surgeons performed radical prostatectomy in lithotomy, 22% in supine, and 3% use a split-leg table (Table 1). When considering predictors of supine positioning; demographic information, years in practice, surgical volume, locale, and training were not associated with positioning. Similarly, access to the Xi platform did not prompt supine positioning. Of the 59 surgeons who used the Xi Robot, 15 (25%) used supine positioning while the remaining majority utilized a lithotomy approach. Those surgeons with access to the Xi model were not significantly different in this preference from their peers using other models (p=0.38). Even among surgeons who use the Xi exclusively, only 36% (10/28) preferred the supine approach, and this, too, was not significantly different from their peers’ practice patterns (p = 0.06).

**Table 1:** Surgeons who use supine versus lithotomy positioning Populations compared with independent sample t-tests, chi-squared tests

Descriptor	N = 92 (%)	Supine	Lithotomy	p-value
Years of Practice (mean)		14.1	13.1	0.69
Robotics Fellowship (n, %)	47 (51)	7 (15)	40 (85)	0.17
Academic Affiliation (n, %)	63 (68)	15 (24)	48 (76)	0.66
Uses Da Vinci Xi^TM ^(n, %)	59 (64)	15 (25)	44 (75)	0.14
Case Volume (n, %)				0.08
<25 cases yearly	28 (30)	10 (36)	18 (64)	
25-50 cases yearly	37 (40)	3 (11)	30 (89)	
>50 cases yearly	27 (29)	7 (19)	24 (81)	
U.S. Location (n, %)	52 (57)	9 (17)	43 (83)	0.36

**Figure 2:**
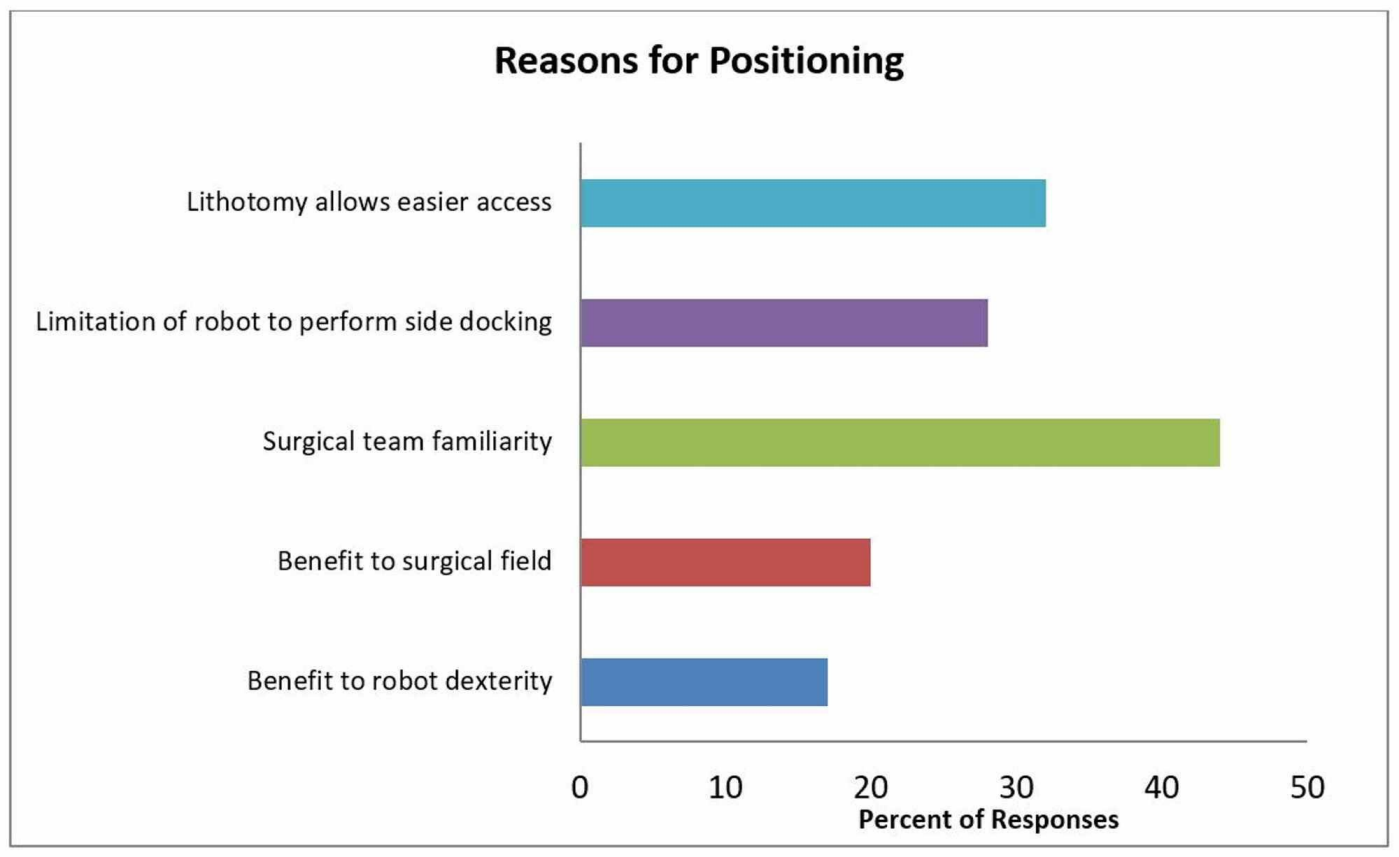
Reasons for lithotomy positioning

Surgeons were asked whether they had considered trialing supine positioning. Fifty (54%) confirmed they had considered it, however, that interest translated to only a minority-19 (38%)-adopting this change. Those most likely to have considered supine positioning were surgeons in the U.S. (69% of U.S. surgeons v. 37% of non-U.S. surgeons, p<0.05) and surgeons who operated using the Da Vinci® Xi™ platform, at least some part of the time (69% of Xi surgeons v. 32% of non-Xi surgeons, p<0.05). The latter result was also noted amongst those surgeons who used the Xi platform exclusively (75%; 21/28 such surgeons, p<0.05). Amongst surgeons with access to the Xi model who had thought about using supine positioning, 65% continued using lithotomy, and only 35% reported switching to the supine approach. Similarly, only 17% of U.S. surgeons performed supine RALP. Notably, a majority of respondents attributed their positioning preferences to their surgical team’s familiarity with lithotomy positioning (Figure 2).

We explored surgeon attributes associated with supine positioning. More respondents without a robotics fellowship (29% v. 15%) and with fewer than 25 yearly RALP cases (36% v. 16%) appeared to favor supine positioning, though this was not statistically significant. Amongst the 57 surgeons who had completed robotic fellowships, those with more years of experience (greater than a median of eight years) were less likely to adopt supine positioning (11% v. 20%, p = 0.4, Fisher Exact Test). No predictors of adopting or considering supine position were found to be statistically significant on multivariate regression, nor when evaluating just the subgroup of surgeons using the Xi robot.

## Discussion

Lithotomy position in steep Trendelenburg has well-established complications, including nerve palsies, compartment syndrome, and rhabdomyolysis, and may thus lead to increased costs [19, 20]. It remains the standard approach to RALP, despite advances in robotics that have allowed for greater flexibility in positioning and operative planning. We report here the first survey of surgeons’ perspectives on RALP positioning. Broadly, surgeons have not been adopting supine positioning. Those who considered or pursued it were more likely to be using the da Vinci® Xi™ platform; however, this increased propensity still summed to only 25% of Xi users. Only 22% of all respondents used supine positioning, and only 54% had considered it. Those continuing to use lithotomy appeared to have higher case volume and may have been more likely to have completed fellowship training. This population cited familiarity in approach for their surgical team as the most common reason for this choice, perhaps reflecting the inertia of surgical training. Given the significant physiologic changes associated with positioning, the modern robotic surgeon should be familiar with positioning options offered by contemporary technologies, and surgeon and surgical-team education efforts should be aimed at overcoming hesitation due to practice-inertia and towards greater creativity.

One should also consider surgeon-education to better understand patterns in positioning preference and as a possible target of intervention. A study by Carrion et al. looked at the urology residency training in Europe by surveying physicians in their final year of training. Those surveyed participated in the European Urology Residents Education Programme (EUREP), which was designed by the European School of Urology (ESU) as a five-day course to standardize resident training [21]. Out of 152 residents, only 26% received robotics training with all receiving 1-10 hours a week and none receiving 11-30 hours [21]. Higher rates of confidence in performing almost all robotics procedures were associated with training in laparoscopy and robotics, participation in practical courses, and having training resources in hospitals [21]. Despite some variation in training patterns between Europe and the U.S., one can extrapolate that more dedicated training may lead to greater confidence in surgical practice and a greater sense of autonomy to try new approaches, including supine positioning.

Such educational differences amongst surgeons may also be reflected in later years of practice. Counterintuitively, our analysis suggests, without statistical significance, that surgeons with more experience, training, and higher case volumes may be less prone to adopting supine positioning over lithotomy. In his analysis of the diffusion of innovation, Rogers described a 5-step process of adoption. The implementation step only occurred after a prolonged information-gathering phase of knowledge acquisition, persuasion, and the decision to try an innovation-all of these are limited by the adopters’ individual qualities and values, as well as those of their institutions [22]. In our study, there was a notable discrepancy between the proportion of surgeons who had considered using supine positioning and those who had actually adopted it-likely reflecting Rogers’ theorized lag from information-gathering to innovation adoption. Mamede et al. show that increasing years of training predicted cognitive anchoring and “availability bias” - the tendency to treat patients similarly to recent similar cases, sometimes without due analysis [23].

Ulmer et al in 2012 found that younger surgeons had been quicker to adopt minimally invasive prostatectomy techniques, commenting that they may have been exposed to more specialized training during residency than their older counterparts [24]. This result was mirrored by Anderson et al.; studying the adoption of minimally invasive prostatectomy during the early 2000s, they commented that surgeons were more likely to adopt and continue novel surgical practices if working with other urologists and trained in those techniques. From a systems perspective, Wright et al. (2011) reported that the adoption of sentinel lymph node biopsy for breast cancer was more avid where high-volume surgeons actively championed the technique, while hospital administrators were more likely to accept variation in practice when presented with guidelines statements to support the change [25]. Perhaps the adoption of supine RALP will lag until a critical mass of surgeons vocally supports or advocates for it to the broader urologic community.

We considered that those who exited training more recently had more exposure to alternative positioning while those further along in their careers may be more established in their approach, potentially correlating with inertia in practice patterns. Our study does not appear to support this hypothesis. Being further in training did not statistically significantly predict respondents’ positioning considerations or choices.

Our study had several limitations. The survey nature of our study may have allowed for selection bias and self-reporting bias. The limited sample size hinders generalizability to a larger population, as might an uneven distribution of international respondents. The strength of this study is that it is the first, if not the only, survey of endourologists concerning position practices in robotic prostatectomy.

The use of lithotomy positioning carries a known risk with position-related complications that have significant effects on surgical outcomes and costs. In supine positioning, there exists a practical and implementable change that can reduce the impact of these negative outcomes. Despite widely used technology being available to facilitate a transition to safer, possibly less-costly, supine RALP, a minority of surgeons appear to have adopted this change. Our survey highlights the need for continuous surgical improvement in light of training-inertia.

## Conclusions

New technologies constantly offer novel advantages, though these may take time to be widely adopted in surgical practice. Current surgical robotics platforms, starting with the da Vinci® Si™ and certainly with the da Vinci® Xi™, allow one to easily obviate the risks associated with lithotomy positioning by performing RALP in supine. A majority of surgeons surveyed continue to perform robotic prostatectomy in lithotomy, although nearly two-thirds have access to the Xi system. Less than half of those who consider using supine positioning actually do. The biggest reason for position choice appears to be the convenience to the surgical team, and this may be related in part to training inertia.

The lag between the availability of new approaches and their widespread use appears to be in line with prevailing theories about innovation adoption, including the ones about new surgical techniques, regardless of their recognized merits. Supine RALP offers considerable advantages for patients and surgeons; its widespread adoption, in line with the aforementioned theories, requires greater communal discussion and local champions to advocate for it. Hopefully, our survey stimulates some of that interest.

## Appendices

Survey sent to members of Endourological Society

1. How many years have you been in practice?

2. Have you had fellowship training?

3. Is your practice in an academic or non-academic setting?

4. Where is your location of practice? (State/Province and Country)

5. How many robotic radical prostatectomies cases do you perform per year?

6. What model of surgical robot do you use for robotic prostatectomies?

7. What positioning do you use most often during robotic prostatectomies? Select all that apply.

     a. Supine

     b. Lithotomy

     c. Other

8. Has supine positioning during robotic radical prostatectomy inhibited your exposure or restricted access to the pelvis in any way? If yes, how has this affected your practice?

9. Have you considered using supine positioning for robotic prostatectomy?

10. What is your reasoning for lithotomy positioning during robotic prostatectomy?

     a. Easy of access

     b. Limitation of robot to perform side docking

     c. Surgical team familiarity

     d. Benefit to surgical field

     e. Benefit to robot dexterity
